# Molecular Mimicry between *Toxoplasma gondii* B-Cell Epitopes and Neurodevelopmental Proteins: An Immunoinformatic Approach

**DOI:** 10.3390/biom14080933

**Published:** 2024-08-01

**Authors:** Karla F. Meza-Sosa, David Valle-Garcia, Hugo González-Conchillos, Tonali Blanco-Ayala, Alelí Salazar, Itamar Flores, Saúl Gómez-Manzo, Dinora Fabiola González Esquivel, Gonzalo Pérez de la Cruz, Benjamín Pineda, Verónica Pérez de la Cruz

**Affiliations:** 1 Neurochemistry and Behavior Laboratory, National Institute of Neurology and Neurosurgery “Manuel Velasco Suárez”, Mexico City 14269, Mexico; karla.meza@innn.edu.mx (K.F.M.-S.); tblanco@innn.edu.mx (T.B.-A.); dinora.gonzalez@innn.edu.mx (D.F.G.E.); 2 Neuroimmunology Department, National Institute of Neurology and Neurosurgery “Manuel Velasco Suárez”, Mexico City 14269, Mexico; david.valle@innn.edu.mx (D.V.-G.); hdgonzalez@innn.edu.mx (H.G.-C.); aleli.salazar@innn.edu.mx (A.S.); ifloresm1903@alumno.ipn.mx (I.F.); 3 Departamento de Inmunología, Escuela Nacional de Ciencias Biológicas, Instituto Politécnico Nacional, Manuel Carpio, Plutarco Elías Calles, Miguel Hidalgo, Mexico City 11350, Mexico; 4 Laboratorio de Bioquímica Genética, Instituto Nacional de Pediatría, Secretaría de Salud, Mexico City 04530, Mexico; saulmanzo@ciencias.unam.mx; 5 Department of Mathematics, Faculty of Sciences, Universidad Nacional Autónoma de Mexico (UNAM), Mexico City 04510, Mexico; gonzalo.perez@ciencias.unam.mx

**Keywords:** molecular mimicry, *T. gondii* infection, pre-gestational immune activation, offspring behavioral and cognitive deficits

## Abstract

Epidemiological studies and meta-analyses have shown a strong association between high seroprevalence of *Toxoplasma gondii* (*T. gondii*) and schizophrenia. Schizophrenic patients showed higher levels of anti-Toxoplasma immunoglobulins M and G (IgM and IgG) when compared to healthy controls. Previously, in a rat model, we demonstrated that the progeny of mothers immunized with *T. gondii* lysates before gestation had behavioral and social impairments during adulthood. Therefore, we suggested that *T. gondii* infection can trigger autoreactivity by molecularly mimicking host brain proteins. Here, we aimed to identify the occurrence of antigenic mimicry between *T. gondii* epitopes and host brain proteins. Using a bioinformatic approach, we predicted *T. gondii RH-88 B* cell epitopes and compared them to human cell-surface proteins involved in brain development and differentiation (BrainS). Five different algorithms for B-cell-epitope prediction were used and compared, resulting in 8584 *T. gondii* epitopes. We then compared *T. gondii* predicted epitopes to BrainS proteins by local sequence alignments using BLASTP. *T. gondii* immunogenic epitopes significantly overlapped with 42 BrainS proteins. Among these overlapping proteins essential for brain development and differentiation, we identified HSP90 and NOTCH receptors as the proteins most likely to be targeted by the maternally generated pathogenic antibodies due to their topological overlap at the extracellular region of their sequence. This analysis highlights the relevance of pregestational clinical surveillance and screening for potential pathogenic anti-*T. gondii* antibodies. It also identifies potential targets for the design of vaccines that could prevent behavioral and cognitive impairments associated with pre-gestational *T. gondii* exposure.

## 1. Introduction

*Toxoplasma gondii* (*T. gondii*) is an obligate intracellular parasite that can infect humans via contaminated food or water [1,2] or by vertical transmission from a pregnant female to her offspring [1]. It is known that *T. gondii* infection during pregnancy can cause severe damage since it can cross the placenta and infect the fetus [1]. In this sense, vertically infected fetuses can present with cerebral calcifications, retinal alterations, micro-, macro- or hydrocephaly, and even spontaneous abortion [3]. Besides gestational defects, congenital toxoplasmosis is known to increase the offspring’s risk of developing autism and different psychiatric disorders characterized by the presence of mental, behavioral, and sociability alterations during adulthood [1,4,5]. Interestingly, epidemiological studies and meta-analyses have shown an association between high seroprevalence of *T. gondii* and schizophrenia [6,7,8,9]. Because most of these patients have already developed schizophrenia, seroprevalence as an indicator of causality remains to be explored. Other studies have also described a correlation between the serological profiles of anti-Toxoplasma immunoglobulins M and G (IgM and IgG) and the occurrence of different neurodevelopmental disorders [10,11,12]. Different studies have shown that the levels of specific maternal pro- and anti-inflammatory cytokines during prenatal infection could either promote or inhibit the development of neurodevelopmental sequelae in offspring [13]. Moreover, a recent study suggested that the inflammatory profile of pregnant women is more important than the actual maternal Th1-type immune response against *T. gondii* in terms of the risk of congenital transmission [3]. In this regard, by using a rat model, we previously showed that the progeny of mothers immunized prior to gestation with *T. gondii* lysates had behavioral and social impairments during adulthood [14]. Interestingly, most models of maternal immune activation (MIA) have explored the role of gestational inflammation in neurodevelopment and its long-term consequences for the offspring [5,15]; however, in our pre-gestational model, inflammation has already resolved at the time the dam is fecundated. Thus, we then explored the possibility that the offsprings’ neurodevelopmental issues were generated by the immune adaptive system, rather than by the innate immune response. Therefore, we suggested the occurrence of molecular mimicry between the parasite and the host brain proteins as a triggering mechanism. According to our hypothesis, *T. gondii* generates an adaptive immune response in which antibodies against *T. gondii* immunogenic peptides are generated. These antibodies can then recognize, albeit with some non-specificity, proteins present in the fetus, particularly those involved in neurodevelopment. Although our previously published experimental data support our hypothesis, the identification of *T. gondii* immunogenic peptides and of which endogenous proteins may be responsible for the molecular-mimicry phenomenon remains to be explored. Thus, in this study, we bioinformatically identified the occurrence of antigenic mimicry by *T. gondii* epitopes of host brain extracellular proteins involved in neurodevelopment. Finally, our results identified molecules that can be used as therapeutic targets in the design of specific vaccines intended to prevent the development of congenital-toxoplasmosis-associated phenotypes. 

## 2. Materials and Methods

For access to the original data, scripts and environment needed to replicate this analysis, visit our GitHub repository: https://github.com/david-valle/2023-toxoplasma_epitopes accessed on 15 June 2024. 

### 2.1. T. gondii Epitope Prediction

First, we obtained all the protein sequences of the *T. gondii* RH88 strain, which are publicly available on the ToxoDB platform (https://toxodb.org/toxo/app accessed on 1 August 2023). A total of 8316 *T. gondii* proteins were analyzed for antigenicity using the standalone Linear B-cell epitope prediction tool from the IEDB analysis resource (http://tools.iedb.org/bcell/ accessed on 1 August 2023). In this analysis, we included five different epitope-prediction algorithms, namely β-turn prediction (Chou-Fasman [16]), Surface accessibility (Emini [17]), Flexibility (Karplus-Schulz [18]), Antigenicity (Kolaskar-Tongaonkar [19]), and Hydrophilicity (Parker [20]). Only epitopes with scores ≥ 1 were considered significant. All epitopes identified by each algorithm that had significant overlap were merged. Then, overlapping epitopes identified by different methods were also merged to obtain a final list of 8584 *T. gondii* peptides. For details about the scripts used in this step, please visit our GitHub repository.

### 2.2. List of Human Surface Proteins Involved in Brain Development and Differentiation

To find human proteins with the potential for molecular mimicry by *T. gondii* B-cell epitopes, we decided to analyze sequence similarity between *T. gondii* epitopes and human proteins. We reasoned that human cell-surface proteins are the most likely to present such similarity, as maternal-derived antibodies would be able to recognize mainly extracellular proteins in the fetus, rather than intracellular ones, the recognition of which would require an active mechanism for antibody internalization. Thus, we decided to focus on human proteins that are annotated as localized on the cell surface according to the Gene Ontology (GO) database (GO:0009986). To confirm that the selected proteins were located on the cell surface, the prediction of extracellular regions was performed using the TOP-CONS and TMHMM2.0 platforms. FASTA sequences were obtained from the Ensembl genes 110 (GRCh38.p14) annotation using Biomart.

A list of human proteins involved in neuron (GO:0030182), astrocyte (GO:0048708), oligodendrocyte (GO:0048709), and microglia (GO:0014004) differentiation according to GO was downloaded through Ensembl’s Biomart. We generated lists of the proteins in each of those categories that overlapped with the list of cell-surface proteins, and the proteins on those lists will be referred to as brain surface proteins (BrainS) from now on. We also generated a list of cell-surface proteins that showed no overlap with any brain-differentiation proteins (named non-BrainS). FASTA sequences of both lists (BrainS and non-BrainS) were downloaded from the Ensembl genes 110 (GRCh38.p14) annotation using Biomart.

### 2.3. Similarity between T. gondii Epitopes and Human Surface Proteins Involved in Brain Development

We generated a protein BLAST database using the BrainS human sequences using the makeblastdb program with default parameters from the BLAST suite. We aligned our predicted *T. gondii* B-cell epitopes to the BrainS database using BLASTP with an e-value cut-off of 0.01. We discarded all alignments with a query coverage below 80%. Our final list of valid alignments was analyzed to obtain the list of significant human protein/gene matches.

### 2.4. Similarity between T. gondii Epitopes and Human Surface Proteins Not Involved in Brain Development

We randomly selected 372 proteins (the same number of proteins in our BrainS dataset) from the non-BrainS list and generated a protein BLAST database using the makeblastdb program with default parameters. We aligned our predicted *T. gondii* B-cell epitopes as described above for the BrainS database, using the same cut-off for e-value and coverage. This process was repeated 100 times, using a random sampling of 372 different non-BrainS proteins each time to obtain a list of “random” non-BrainS matches.

### 2.5. Protein Network of Human Proteins with the Potential for Molecular Mimicry 

To generate a protein-protein interaction network, we used the STRING database (https://string-db.org/ accessed on 1 August 2023) and included only our candidate proteins. Proteins are connected by edges, which indicate either an experimentally validated physical interaction or a predicted interaction based on the co-expression data of the connected proteins. The network was generated using high confidence (0.700). Later, a Gene Ontology (GO) analysis was performed, and proteins involved in neurogenesis, axonogenesis and gliogenesis were labeled in red (false discovery rate (FDR) of 1.45 × 10^−15^), purple (FDR of 8.36 × 10^−15^), and yellow (FDR of 1.57 × 10^−5^), respectively.

### 2.6. Expression of Genes Identified as Potential Targets of Molecular Mimicry in Mouse Development

Expression levels for all predicted genes were downloaded from ENCODE (ENCODE v1.2.1 mm10-M21 version). We used the available forebrain, midbrain, and hindbrain datasets from mouse embryonic (E) days E10.5, E11.5, E12.5, E13.5, E14.5, E15.5, and E16.5. Z-scores were calculated for each gene and plotted in a heatmap in R using heatmap.2 from the gplots package.

### 2.7. Prediction of Protein Topology

The best candidate BrainS proteins were selected based on the statistical significance (e-value) of their alignment against *T. gondii* epitopes. The e-value represents the probability that a specific alignment is due to a true biological relationship and not chance. An e-value lower than 0.1 was used as the cut-off for this analysis. The lower the e-value, the more significant the alignment is, as it suggests a higher probability that the sequences share a common evolutionary origin. Thus, the selected proteins were those with the lowest e-values. Then, to identify the amino acid (aa) sequences corresponding to the extracellular regions of the best candidates, the sequences of the later ones were obtained. and their topologies were predicted by using both the TMHMM 2.0 prediction tool (https://services.healthtech.dtu.dk/services/TMHMM-2.0/ accessed on 17 October 2023) [21] and the TOPCONS platform (https://topcons.cbr.su.se/ accessed on 24 October 2023) [22]. 

### 2.8. Overlapping of Protein Structures

Based on the previous step, tridimensional overlapping was carried out only for those alignments that yielded an e-value lower than 0.1. Then, the structures of both the BrainS and *T. gondii* proteins were searched and downloaded in the pdb format from the AlphaFold database (https://alphafold.ebi.ac.uk/ accessed on 17 October 2023) [23,24]. These files were analyzed with ChimeraX software version 1.6 [25]. Finally, the extracellular regions of BrainS proteins were identified and the sequences of the *T. gondii* epitopes that aligned with the best candidates were superimposed.

## 3. Results

### 3.1. Prediction of T. gondii B-Cell Epitopes

**Figure 1:**
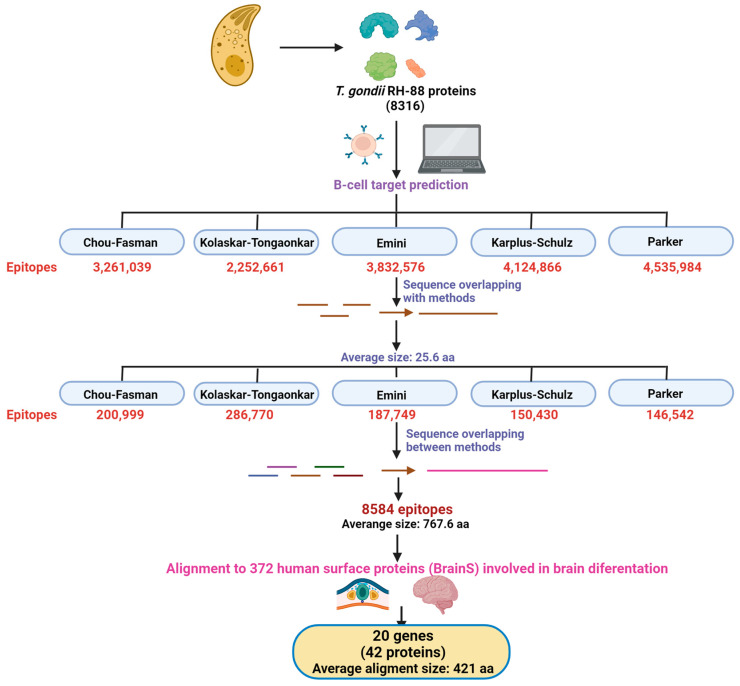
Analysis scheme: A global scheme of our bioinformatic pipeline is shown. Briefly, potential epitopes from *Toxoplasma gondii* (*T. gondii*) proteins were predicted bioinformatically. Epitopes were merged within and between prediction methods. Matches from those epitopes with human surface proteins expressed during brain differentiation (neurodevelopment) were searched. Transmembranal regions for those matching proteins were predicted to find high-confidence predictions of molecular mimicry. aa—amino acids.

To determine whether molecular mimicry occurs between human brain proteins and *T. gondii* B-cell epitopes, prediction of linear B-cell epitopes was performed on the 8316 available protein sequences of the RH-88 *T. gondii* strain. Five different algorithms that considered β-turn regions, surface accessibility, flexibility, antigenicity, and hydrophilicity were used (Figure 1). Only peptides with significant scores were considered. Following this methodology, 2–4 million short epitopes of about seven amino acids each, depending on the algorithm, were predicted. To consolidate the number of predicted peptides, we stitched together those peptides with overlapping positions on the same protein, first merging the peptides predicted for each algorithm. This increased the average size of our predicted epitopes to ~25.6 aa and dramatically reduced the number of epitopes to 150–280 thousand peptides per algorithm. Finally, the epitopes with overlapping positions were merged onto a single protein, pooling the peptides from all algorithms. We then merged redundant epitopes with the same sequence, even if they were found in independent proteins. This gave us a total of 8584 *T. gondii* epitopes with an average size of 767.6 aa that are predicted to elicit the response of B-cells and thus potentially generate antibodies against them (Figure 1, Supplemental S1). Although most of these epitopes still lack experimental confirmation, we used this high-confidence prediction for further analysis.

### 3.2. Prediction of Human Proteins with Potential for Molecular Mimicry by T. gondii B-Cell Epitopes

Because we previously observed behavioral changes in progeny derived from mothers exposed pre-gestationally to *T. gondii* lysates [14], we hypothesized that these phenotypes may be caused by neurodevelopmental defects caused by the aberrant binding of maternally provided antibodies that originally recognized *T. gondii* proteins to the extracellular domains of proteins involved in the fetus’s nervous-system development. Thus, we decided to focus on looking for human cell-surface proteins involved in brain development and differentiation processes with a high degree of similarity to *T. gondii* epitopes. To do so, from the obtained list of cell-surface proteins, we searched the ones annotated, according to GO, as being part of the differentiation processes of the main cell populations that make up the nervous system: neurons, astrocytes, oligodendrocytes, and microglia cells. As expected, most of these proteins were related to the neuronal lineage, with the second-most common being astrocytic proteins, then, in similar proportion, proteins of the microglial and the oligodendrocytic lineages. This set of 372 surface proteins was called the “BrainS” proteins, as they are involved in the differentiation of distinct cell types within the nervous system (Figure 1).

Then, the *T. gondii* predicted epitopes were compared to the BrainS proteins by local sequence alignments using BLASTP. For this purpose, high-coverage alignments (>80% coverage) with a significant e-value (<0.01) were considered (Supplemental S2). Using those parameters, we detected 42 proteins that aligned with *T. gondii* epitopes. Those 42 proteins correspond to only 20 genes (as many of them are isoforms generated from the same gene) and are summarized in Table 1**.** This set of proteins was referred to as “the potential molecular-mimicry proteins”.

**Figure 2:**
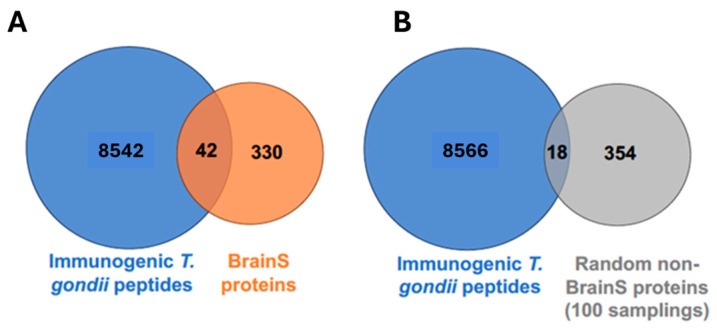
Human cell-surface proteins matching *T. gondii* epitopes. (**A**) Venn diagram showing the overlap between *T. gondii* epitopes (blue, left) and human cell-surface proteins involved in neurodevelopment (BrainS proteins; orange, right). (**B**) Venn diagram showing the average overlap between *T. gondii* epitopes (blue, left) and randomly selected human cell-surface proteins not involved in neurodevelopment (non-BrainS proteins; gray, right). The random selection was repeated 100 times, as depicted.

Although we were cautious in using high-stringency parameters in both the epitope prediction and the sequence alignment, given the high number of epitopes, we wondered whether the observed overlap of 42 proteins was likely to be found by chance using our pipeline. To answer that question, we reasoned that if there is something particular about the BrainS proteins, a set of non-BrainS proteins would not be likely to give us as many hits. So, we eliminated the BrainS proteins from our pool of cell-surface proteins and generated a new dataset that we called non-BrainS, which included all cell-surface proteins not annotated as being involved in brain development. We randomly sampled 372 proteins from the non-BrainS pool (to make a fair comparison to our 372 BrainS proteins) and repeated the alignment against *T. gondii* epitopes, using the same parameters. The random sampling/alignment was repeated 100 times. This means that each time, 372 different non-BrainS proteins were sampled and aligned against *T. gondii* epitopes. On average, the 372 randomly sampled non-BrainS proteins had just 18 matches with *T. gondii* epitopes, in sharp comparison to the 42 hits found using the BrainS dataset (Figure 2). This difference is statistically significant (*p*-value < 0.0001), suggesting that the overlap between BrainS and *T. gondii* epitopes is not due to chance. This supports the hypothesis that higher similarity should be seen when cell-surface proteins involved in brain development are considered than when cell-surface proteins that are not involved in brain development are considered. This may not be so surprising if we consider that *T. gondii* has a natural tropism for brain tissue, but it is in line with what we expected according to our hypothesis.

**Figure 3:**
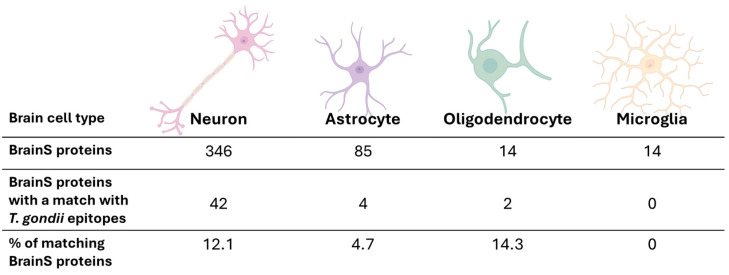
Proteins matching *T. gondii* epitopes by cell type. In the second row, the total number of proteins in the BrainS dataset is indicated for each cell type. The next row shows the number of proteins in the BrainS dataset that matched *T. gondii* epitope(s) for the indicated cell types. The percentage of matches compared to the total number of proteins is shown at the bottom row.

Then, the list of human proteins with the potential for molecular mimicry by *T. gondii* epitopes was analyzed carefully. In particular, the protein list was subdivided based on cell type (neuron, astrocyte, oligodendrocyte, or microglial cell). Most of the proteins are annotated as neuronal (Figure 3), which may be due to a bias in research and annotation. Among all 346 neuronal annotated proteins, 42 (~12%) (Figure 3), which were generated from 20 genes, were included in our list of potential targets of molecular mimicry (Table 1). For astrocytes, the second-most-common category, only ~5% of proteins (ROR1, ROR2, NOTCH1 receptor and EPHA4) have the potential for molecular mimicry. Interestingly, ~14% of oligodendrocyte proteins (LYN and NOTCH1 receptor) have the potential for molecular mimicry, while none of the microglial proteins have matches with *T. gondii* epitopes (Figure 3). Although our results must be considered carefully given the differences in protein number between cell types, this would suggest that the cells that would be most affected by maternal antibodies during the progeny’s neurodevelopment neurons and oligodendrocytes.

### 3.3. Network Prediction of Human Proteins with the Potential for Molecular Mimicry

**Figure 4:**
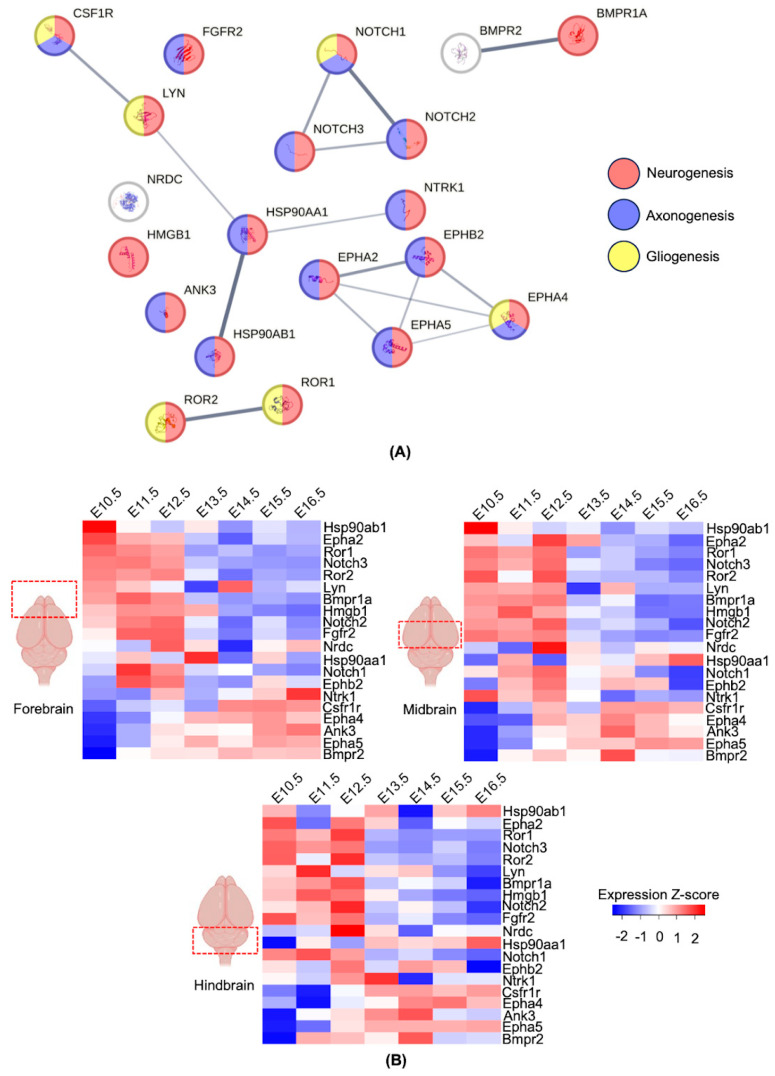
Protein–protein interaction network and expression profiles of genes encoding human proteins with the potential for molecular mimicry. (**A**) Protein–protein interaction network of identified candidates. The ball color indicates whether they are involved in neurogenesis (red), axonogenesis (blue-purpleish) or gliogenesis (yellow) according to Gene Ontology (GO). Edge thickness between proteins indicates the level of confidence for that interaction. (**B**) Heatmaps showing normalized expression levels of the 20 identified candidates at the transcript level (messenger RNA) in three mouse-brain regions (forebrain, midbrain, and hindbrain) at different embryonic (E) days, which are indicated above each column.

To get insight into the possible molecular mechanism by which maternal-derived antibodies may be affecting offspring’ behavior during the adulthood, the next question was whether the potential identified mimotopes were co-related, whether they had similar function(s), or whether they were independent. To answer this question, first, the publicly available Search Tool for the Retrieval of Interacting Genes/Proteins (STRING) was used to analyze only reported and experimentally validated interactions between potential mimotopes. As shown in Figure 4A, most of the proteins are linked. Interestingly, 3 small protein clusters were found: Cluster 1, composed of NOTCH receptor proteins; Cluster 2, composed of EPH proteins; and Cluster 3, composed of LYN, CSF1R, NTRK1 and two HSP proteins. As a result, these analyses indicated three possible mechanisms of action that must be experimentally explored (see Section 4 for details). As expected, GO analysis showed that most of the proteins are involved in neurogenesis, axonogenesis and gliogenesis. The fact that most of these proteins participate in axon growth and guidance would suggest that the offspring of mothers exposed to *T. gondii* may show defects in axon biogenesis. Indeed, this is a frequently altered process, as reported in different in vitro and in vivo models of schizophrenia [26,27,28], among other neurodevelopmental disorders.

### 3.4. Expression Profiles of the Genes with the Potential for Molecular Mimicry during Development

Finally, the expression patterns of the proteins in our potential-molecular-mimicry dataset were analyzed using publicly available databases (see Section 2 for details, Supplemental S3). It was found that around two thirds of our identified candidates have an early expression pattern (i.e., their highest expression level is between embryonic (E) days E10.5–E12.5) in all analyzed mouse-brain areas: forebrain, midbrain, and hindbrain (Figure 4B). On the other hand, one third of our candidates showed higher expression levels at later time points (E14.5–E16.5). This pattern was observed in the three analyzed brain regions. As most of the candidates fulfill functions of differentiation and polarization of cells in the nervous system (Table 2), the main effect of the mimicry mechanism would be to affect early brain development, which would explain the lasting effects we observed experimentally.

### 3.5. Structural Overlap in Extracellular Regions

**Figure 5:**
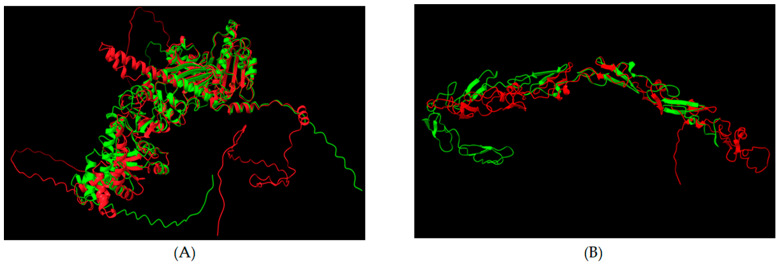
Prediction of protein topology and overlap with *T. gondii*. (**A**) Structural overlap between human heat shock protein 90 (HSP90AA1 or HSP90α, green) and *T. gondii* putative HSP90 protein (red). (**B**) Overlap between human NOTCH1 receptor protein (green) and *T. gondii* EGF-Like domain-containing protein (red). The extracellular regions of BrainS proteins (HSP90AA1 and NOTCH receptor) are highlighted in green, while transmembrane and intracellular regions were removed.

Although the sequence or structural similarities between candidate BrainS proteins and *T. gondii* epitopes would indicate a possible immune-system response due to molecular mimicry, it is necessary to consider the topology of the BrainS proteins since, even if this similarity exists, if the shared region is not accessible for recognition, activation of an immune response will not take place. By predicting the topology of BrainS proteins, we were able to identify which candidates have sequence identity to *T. gondii* peptides in their extracellular region. Of the 20 candidate proteins, 6 (HSP90α, HSP90β, NRDC, HMGB1, LYN and ANK3) were predicted to be non-cytoplasmic proteins. According to the GO database and topology-prediction data, the cell-surface protein HSP90α is the candidate with the highest probability of exhibiting high similarity to the putative HSP90 protein of *T. gondii* (Figure 5A). Moreover, the NOTCH1 receptor (Figure 5B), two other NOTCH-receptor isoforms (2 and 3), and ROR1 present structural overlap in their extracellular segments with *T. gondii* proteins. As to the remaining 10 candidate BrainS proteins, the segments presenting similarity to *T. gondii* peptides were predicted to be transmembrane or cytoplasmic, so they could not be recognized by autoantibodies and would be less likely to be associated with molecular mimicry. 

## 4. Discussion

*Toxoplasma gondii* (*T. gondii*) is an obligate intracellular protozoan parasite that causes toxoplasmosis. It has been estimated that 30% of the global human population is chronically infected with *T. gondii*, which is why the parasite is considered a world health problem [45,46]. It has been extensively described that infectious agents can cause psychiatric disorders when they infect adults. In this context, epidemiological studies and meta-analyses have consistently supported a link between *T. gondii* infection and schizophrenia [47,48] and between *T. gondii* infection and other neuropsychiatric disorders such as bipolar disorder [49,50] and obsessive-compulsive disorder [49,51]. These findings have prompted interest in elucidating whether this infectious agent can cause schizophrenia or schizophrenia-like diseases. Moreover, it remains unknown whether this causal relationship between *T. gondii* infection and schizophrenia is favored depending on whether the infection occurs in a developing fetus, a newborn, or a young child [52]. Another possible source of variability would be whether the infection happened before or during early pregnancy, since it was demonstrated that maternal seropositivity to anti-*T. gondii* IgGs increases the risk of a psychiatric disorder developing in their offspring [12]. This association was later supported by another study wherein subjects with a psychiatric disorder showed seropositivity to anti-*T. gondii* IgG at birth [52]. 

A characteristic shared by both studies was the presence of IgG and the lack of IgM seropositivity, indicating that the infection was not recent; this was in addition to the absence of pro-inflammatory cytokines. Thus, these findings together suggest an autoreactivity process triggered by *T. gondii*. It has been described that *T. gondii* infection can trigger autoreactivity by different mechanisms. Here, we proposed that autoreactivity is due to molecular mimicry of host proteins [53]. In a previous experimental study, we demonstrated that IgG antibodies against *T. gondii* from maternal serum recognize and bind to proteins found in fetal brain tissue [14]. Therefore, we suggested that this observed cross-reactivity could be induced by molecular mimicry by parasite antigens sharing a large percentage of identity. For example, the *T. gondii* HSP70 protein shares an identity of 76% with its human orthologue [53]. Thus, in our pregestational model, autoreactivity could be a determining factor affecting binding to key neurodevelopmental proteins, which leads to their malfunction and possibly alters neuronal physiology, including neural circuit formation and neurotransmission.

In our prior experimental study, the possibility that both cognitive and neurotransmission alterations were due to the presence of the living parasite or because of a maternal inflammatory response was discarded via tests using a *T. gondii* lysate. As expected, we found that those rats immunized with the *T. gondii* lysate produced anti-*T. gondii* antibodies capable of binding to 18-day-old fetal brain proteins. Moreover, IgG antibodies were found to bind to fetal brain structures in the offspring of female rats that had been immunized with *T. gondii* lysate before gestation [14]. However, the identity of these proteins and the specific mechanism by which they were being recognized by maternal anti-*T. gondii* antibodies were open questions.

Consequently, here we aimed to identify those *T. gondii* epitopes that share high identity with key neurodevelopmental proteins essential for the proper formation of neuronal circuits and neurotransmission function during gestation and early life. Through a bioinformatic pipeline we designed (see Section 2 for details), we were able to identify a total of 8584 *T. gondii* epitopes with a high probability of being recognized by B-cells and inducing them to produce antibodies (Figure 1, Supplemental S1). Then, we analyzed sequence similarity between the 8584 identified *T. gondii* epitopes and human cell-surface proteins (372 proteins according to their GO) involved in neurodevelopment and differentiation processes that, due to their cellular location, would have a higher probability of being recognized by maternal antibodies in the fetal brain. When the alignment was made, 42 proteins were identified as potential targets of mimicry, being many of them isoforms of the same gene (20 genes, Table 1). Among the proteins with high-coverage alignments (>80% coverage) are ANK3, BMPR1A, BMPR2, CSF1R, EPHA2, EPHA4, EPHA5, EPHB2, FGFR2, HMGB1, HSP90AA1, HSP90AB1, LYN, the NOTCH1 receptor, the NOTCH2 receptor, the NOTCH3 receptor, NRDC, NTRK1, and ROR1 and ROR2. As expected, the 42 proteins were related to the neuronal lineage, since the first selection criterion was to include human surface proteins involved in neurodevelopment and CNS differentiation processes. Notably, these 42 proteins represented 12.1% of the 346 analyzed neuronal proteins. In contrast, oligodendrocytes, with only 14 analyzed proteins, had 2 matches, thus representing 14.3% of the analyzed proteins and having a higher probability of being targeted for molecular mimicry by *T. gondii* epitopes. Astrocytes, wi6u 85 analyzed proteins, were represented in 4.7% of matches, and microglial proteins did not generate any matches.

Most of these proteins are involved in essential neurodevelopmental processes such as neurite growth, neural differentiation, and polarization. Upon examining the relationships between these proteins, we found three clustering groups: (1) NOTCH receptor proteins, which are highly conserved across species, with at least two rounds of gene duplication in mammals (NOTCH1-4 receptors) and are vital in developmental cell-fate decisions. In the nervous system, the NOTCH signaling pathway regulates the transcription of several genes including *hes1*/*5*, the HES- related genes (*hesr1*/*2*), the glial fibrillary acidic protein (*gfap*) and the brain lipid-binding protein (*blbp*) [54]. The NOTCH1 and NOTCH3 receptors are expressed in mammalian spinal-cord precursor cells and immature neurons and are important for neuronal differentiation and maturation of the spinal cord [55]. Moreover, NOTCH receptor signaling maintains NSCs in a proliferative state [56], which is essential for generating the optimal number of neurons necessary for the formation of neural circuits. However, the inhibition of NOTCH receptor signaling in the outer region of the subventricular zone is crucial to the induction of neuronal differentiation of NSCs [57,58]. The NOTCH receptor is also implicated in gliogenesis independently of its effects on progenitor maintenance; hence, NOTCH receptor signaling initially promotes neural-progenitor generation and subsequently promotes gliogenesis [59]. Thus, a fine balance between inhibiting and activating the NOTCH receptor signaling pathway is essential for the early stages of neurodevelopment [60]. In addition, in a genome-wide molecular-network analysis, NOTCH1 was predicted to be one of the most important nodes that link genes in which genetic variants possibly involved in schizophrenia susceptibility due the presence of *T. gondii* seropositivity [61]. Additionally, BMPs inhibit neuronal differentiation and promote glial differentiation during embryonic neurogenesis. BMP signaling appears to work in parallel with NOTCH receptor signaling to maintain NSCs without being redundant [60]. (2) The second cluster consisted of EPHRIN (EPH) receptors, which are essential for regulating the formation of retinotopic maps during development; these allow visual images to be transferred spatially intact [62]. Complementary gradients of EPH receptors on the axons of retinal ganglion cells and ligands within the tectum are thought to mediate the formation of topographical connections. Activation of EPH signaling in retinal ganglion cells reduces cell adhesion and leads to growth-cone collapse [63]. Furthermore, the EPHB receptor has been implicated in the establishment of neural pathways. The dynamic and tightly regulated spatio-temporal expression patterns of EPHB2 and its ligands are associated with the development of the olfactory system [36,64]. A third cluster includes LYN, CSF1R, NTRK1 and HSP90, all of which are involved in neurogenesis, axogenesis, and gliogenesis processes. For instance, HSP90, which is highly expressed during the G0–G1 phase of neural-plate induction, maintains constant expression and maintains its subcellular localization in Purkinje cells throughout the postnatal period and into adulthood. It has been suggested that HSP90 contributes to neurite outgrowth and modulates cytoskeleton dynamics and neuronal polarization [36,65]. 

In summary, immune cross-reactions with at least one of these key proteins for neurodevelopment would jeopardize the orchestration of multiple neurobiological processes, mainly neurogenesis and neuroplasticity. Such disruptions may manifest in adults as cognitive deficits such as those previously found in our experimental study. The challenge for future studies is to corroborate that these bioinformatic targets are the mechanistic link responsible for the cognitive and behavioral impairments characteristic of neuropsychiatric disorders such as schizophrenia. In this context, we are currently experimentally determining which of the proteins we identified as putative targets of molecular mimicry are indeed recognized by maternally generated anti-*T. gondii* antibodies in vitro. Once we identify the true targets of molecular mimicry, the next step will consist of designing vaccines that prevent binding between maternally generated anti-*T. gondii* antibodies and these proteins, which are key for neurodevelopment. Therefore, these vaccines would reduce the offspring´s risk of presenting congenital toxoplasmosis-associated phenotypes with social and mental consequences.

## 5. Conclusions

In this study, we identified numerous antigens derived from *Toxoplasma gondii* RH88 that exhibit molecular mimicry of human proteins involved in neurodevelopment. This represents the first report linking anti-*T. gondii* IgG antibodies with specific human proteins identified as key in early brain development. As mentioned in the discussion, future research should focus on determining whether binding to these target proteins by pathogenic anti- *T. gondii* maternal antibodies is a causal molecular mechanism that could explain the neuropathological sequelae in the offspring following maternal exposure to *T. gondii*. Moreover, the putative target proteins generated from this analysis highlight the relevance of pre- and post-gestational clinical surveillance, especially in a context in which we have experienced contact with infectious agents, for example, the COVID-19 pandemic. In addition, these results could be valuable in future clinical and basic research and could support the development of a screening for potentially pathogenic anti-*T. gondii* IgG antibodies in women prior to pregnancy (pre-gestational screening) and, as previously mentioned, support the design of vaccines that prevent the binding of pathogenic IgG antibodies generated by exposure to *T. gondii*.

## Figures and Tables

**Table 1:** List of human cell-surface proteins involved in neurodevelopment and matching *T. gondii* epitopes.

	Alignment Positions				Extracellular Region Positions	
Protein Name	Start (aa)	End (aa)	Alignment Length	e-Value	Start (aa)	End (aa)
ANK3	363	527	168	9.09 × 10^−16^	φ	φ
BMPR1A	240	529	321	6.20 × 10^−11^	24	153
BMPR2	206	534	354	3.78 × 10^−7^	27	149
CSF1R	704	908	213	3.48 × 10^−9^	22	515
EPHA2	615	870	264	3.01 × 10^−17^	24	535
EPHA4	622	876	263	6.30 × 10^−15^	22	547
EPHA5	694	971	327	3.75 × 10^−13^	22	573
EPHB2	625	877	261	6.40 × 10^−17^	22	544
FGFR2	399	666	272	3.64 × 10^−14^	23	377
HMGB1	83	165	85	2.70 × 10^−12^	φ	φ
HSP90AA1	18	703	721	0.0	φ	φ
HSP90AB1	17	717	739	0.0	φ	φ
LYN	248	508	284	2.65 × 10^−8^	φ	φ
NOTCH1 receptor	707	944	283	1.28 × 10^−4^	22	1735
NOTCH2 receptor	651	922	281	6.31 × 10^−8^	26	1677
NOTCH3 receptor	1079	1373	295	7.30 × 10^−7^	22	1643
NRDC	205	1089	916	2.52 × 10^−65^	φ	φ
NTRK1	509	777	273	6.65 × 10^−11^	31	417
ROR1	476	736	262	9.72 × 10^−15^	30	406
ROR2	476	738	264	1.59 × 10^−20^	31	405

The table shows the 20 longest significant alignments between *T. gondii* epitopes and human proteins. The “alignment position” column shows the positions of both the first and the last amino acids (aa) of each BrainS protein that aligned with the *T. gondii* epitope. The “extracellular region” column shows the positions, for each BrainS protein, of both the first and the last aa of that are predicted to be exposed at the cell surface. Coordinates are based on human proteins (BrainS). φ: the prediction indicates that the topology of the protein is extracellular. aa: amino acids.

**Table 2:** Functions of molecular mimicry candidate proteins.

Candidate	Function	References
ANK3	Ankyrin-3 (ANK3) regulates dendrites morphology and N-methyl-D-aspartate (NMDA) receptor trafficking. ANK3 participates in the formation and maintenance of the axon initial segment (AIS) and the nodes of Ranvier. Some mutant variants of the *ANK3* gene have been associated with neurological disorders such as schizophrenia and autism.	[29,30]
BMPR1A/BMPR2	Bone morphogenetic protein receptor type 1A (BMPR1A) and type 2 (BMPR2) are receptors for the bone morphogenetic proteins (BMPs). The BMP signaling pathway induces the formation of the dorso-ventral axis of the developing spinal cord and brain, neurogenesis, and later astrogliogenesis, and it participates in neurite outgrowth from immature neurons. Inhibition of BMP signaling during early development promotes neuroectoderm-from-ectoderm differentiation.	[31]
CSF1R	Colony-stimulating factor 1 receptor (CSF1R) is the receptor for the colony-stimulating factor 1 (CSF1) cytokine, which controls the generation, differentiation, and function of macrophages. In the central nervous system (CNS), CSF1 is a major regulator of microglial development and maintenance. Meanwhile, CSF1R is expressed in neural progenitor cells (NPCs). Mice lacking CSF1R exhibit global defects in brain development, including atrophy of the olfactory bulb, expansion of the lateral ventricle, thinning of the neocortex, and functional abnormalities of the sensory nervous system.	[32]
EPHA2-5/EPHB2	The erythropoietin-producing hepatocellular (EPH) family of tyrosine kinases receptors (RTKs) includes several members. EPH-B2 is expressed in hippocampal astrocytes, and it regulates neurogenesis and promotes neuronal differentiation of neural stem cells (NSCs). These receptors regulate cell migration by promoting changes in cellular adhesion to the extracellular matrix. They are also involved in the remodeling of efferent axons to ensure the correct and precise innervations to their target cells, in dendritic spine morphology, in synaptogenesis, and in synapse stabilization.	[33]
FGFR2	Fibroblast growth factor receptor 2 (FGFR2) controls neuronal morphological maturation, neuronal migration, and spine density during cortical development by interacting with the neuronal growth regulator 1 (NEGR1) protein.	[34]
HHMGB1	High mobility group box 1 (HHGB1) is required for the differentiation and proliferation of NSCs and NPCs. It also promotes neurite outgrowth during early forebrain development.	[35]
HSP90AA1 (HSP90α)	Heat shock protein 90 alpha family class A member 1 (HSP90AA1) is induced by stress and can be expressed in the brain, retina, and spinal cord. It is highly expressed between the G0 and G1 cell cycle phases during embryonic neurodevelopment which correlate with neuronal differentiation and polarization. It also promotes neurite growth.	[36,37]
HSP90AB1 (HSP90β)	Heat shock protein 90 alpha family class B member 1 (HSP90AB1) is highly and constitutively expressed in the brain, the retina, and the spinal cord between the G0 and the G1 phases of the cell cycle during embryonic neurodevelopment. It stabilizes and controls the activity of hypoxia inducible factor-1 (HIF-1) to promote NPC proliferation.	[38]
LYN	*Lyn* expression is relatively low in the brains of embryonic and neonatal mice. However, at the protein level, LYN has been suggested to participate in the formation and function of synapses between granule and Purkinje cells in the rat cerebellum, resulting in motor learning.	[39,40]
NOTCH1-3 receptor	The NOTCH receptor signaling pathway keeps NSCs in a proliferative state by promoting their survival and self-renewal. This pathway also has a role in defining cell fate (neuronal or glial). Briefly, it induces the expression of HES1 and HES5, which repress the expression of proneural genes and therefore inhibit neural differentiation.	[41]
NRDC	Nardilysin convertase (NRDC) is a regulator of axonal maturation and myelination of both the CNS and the peripherical nervous system (PNS). Axon diameter and myelin thickness correlate with NRDC expression levels. It also participates in the proteolysis of NGR1, which is a regulator of myelination.	[42]
NTRK1	Neurotrophic receptor tyrosine kinase type 1 (NTRK1), also known as TRKA, belongs to a family of nerve growth-factor receptors whose ligands include neurotrophins, like the nerve growth factor (NGF), which is involved in the regulation of the development of central and peripheral neurons.	[43]
ROR1-2	Receptor tyrosine kinase-like orphan receptors (RORs) 1 and 2 are expressed exclusively in the developing nervous system, including in the mouse neocortex. It also regulates neurite extension and synapse formation in hippocampal neurons. The ROR1-WNT5a and ROR2-WNT5a signaling pathways regulate the maintenance of the proliferative and neurogenic states of NPCs.	[44]

## Data Availability

The data presented in this study are available on request from the corresponding author.

## Supplementary Materials

The following supporting information can be downloaded at: https://www.mdpi.com/article/10.3390/biom14080933/s1, Supplemental S1: FASTA genes, Supplemental S2: PDBs Match, Random, sequences, Supplemental S3: Brain protein expression.
