# Supplementary figures and images for: Molecular Mimicry between Toxoplasma gondii B-Cell Epitopes and Neurodevelopmental Proteins: An Immunoinformatic Approach
Source: Biomolecules. 2024 Aug 1;14(8):933. doi: 10.3390/biom14080933 (PMC11352964; doi:10.3390/biom14080933)

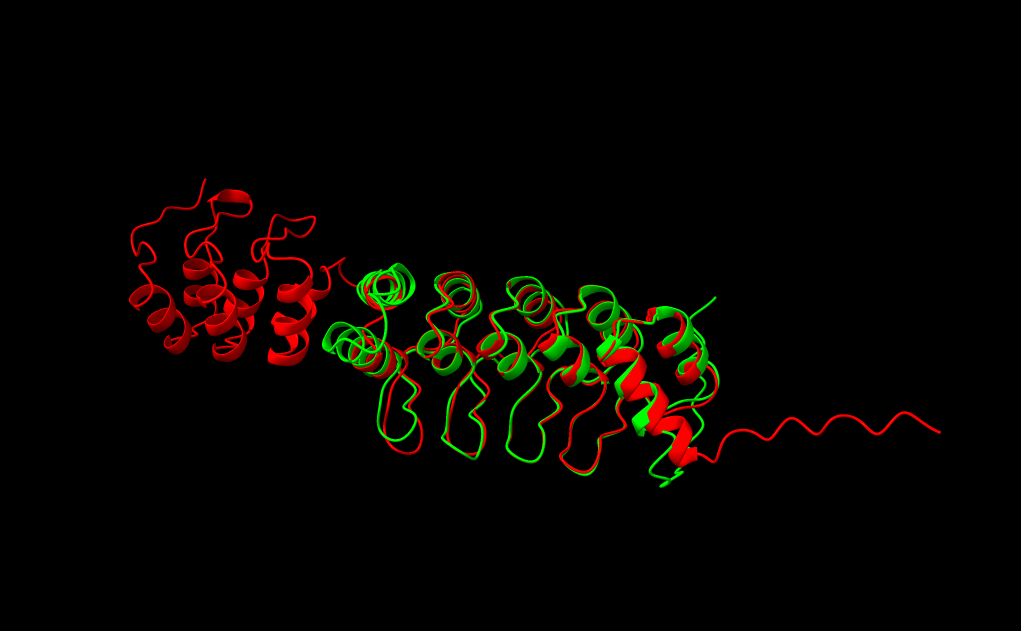

Supplement: Supplementary file 1 [file biomolecules-14-00933-s001.zip › Supplemental S2/PDBs_Match/ANK3.png]

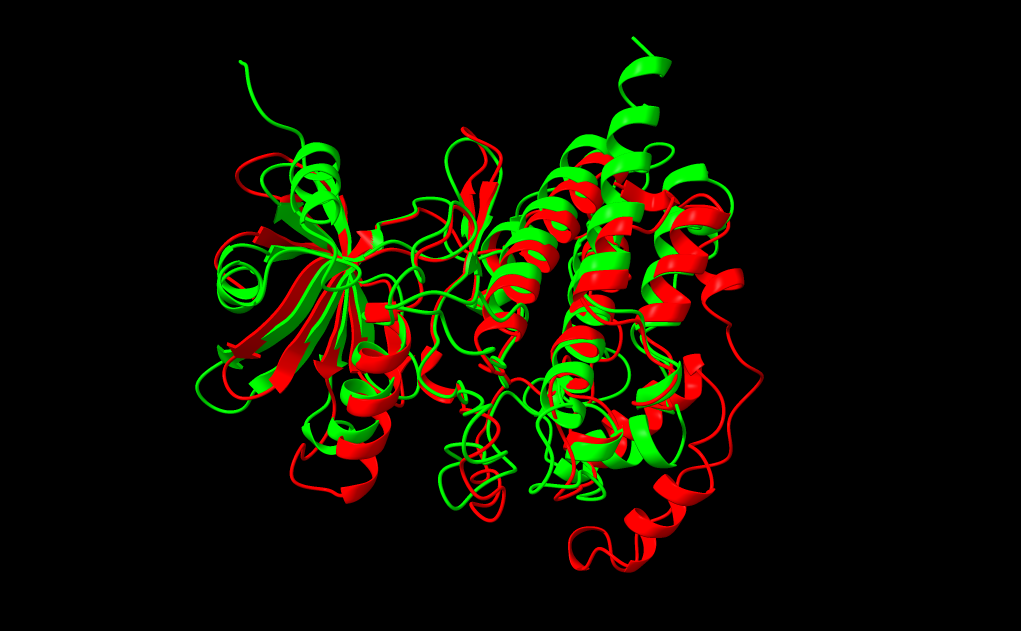

Supplement: Supplementary file 1 [file biomolecules-14-00933-s001.zip › Supplemental S2/PDBs_Match/BMPR1A.png]

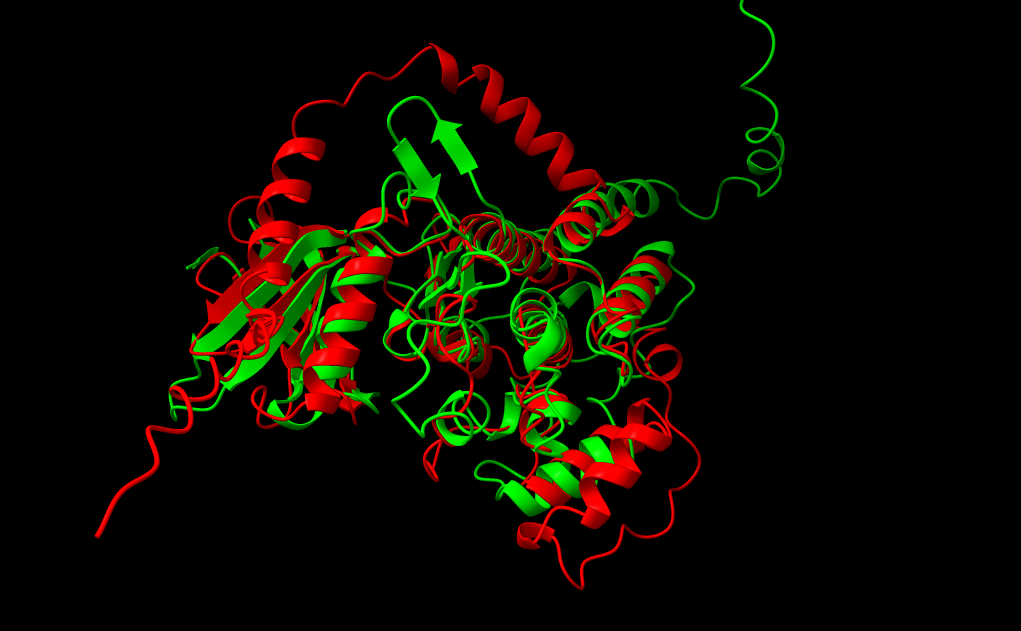

Supplement: Supplementary file 1 [file biomolecules-14-00933-s001.zip › Supplemental S2/PDBs_Match/BMPR2.png]

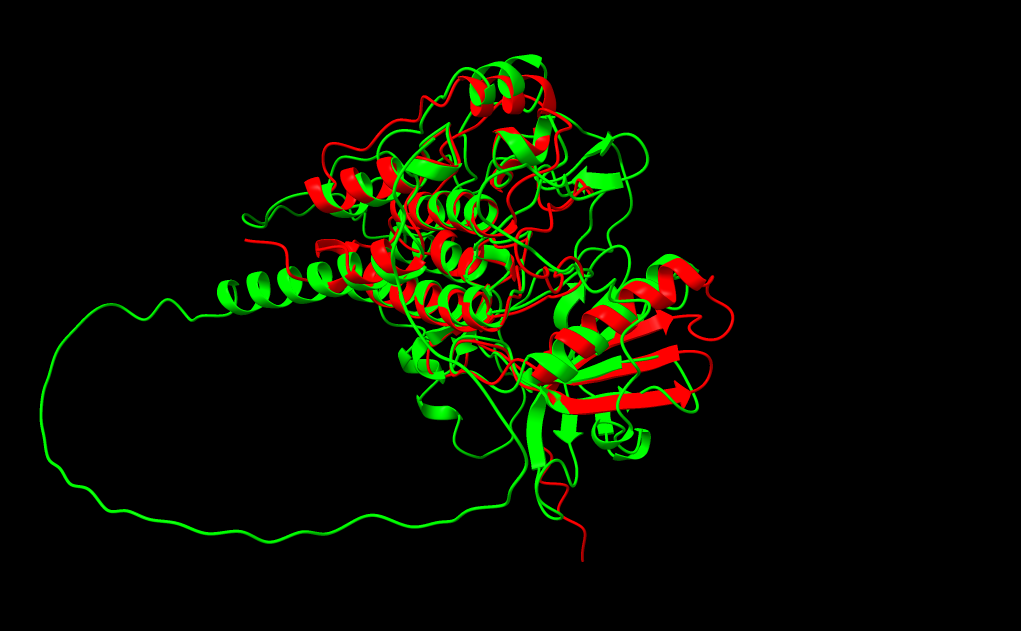

Supplement: Supplementary file 1 [file biomolecules-14-00933-s001.zip › Supplemental S2/PDBs_Match/CSF1R.png]

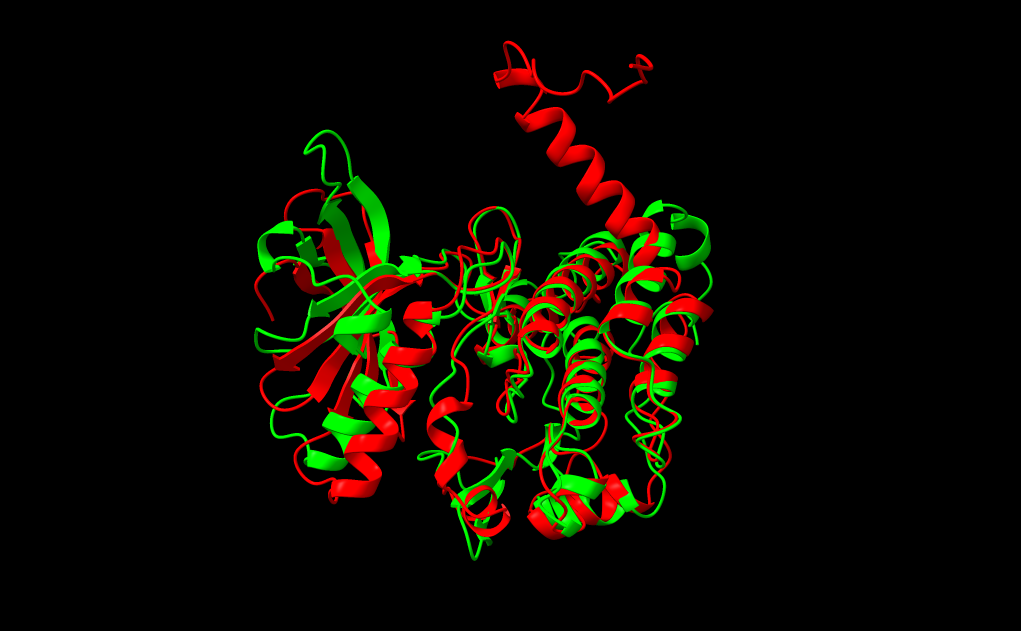

Supplement: Supplementary file 1 [file biomolecules-14-00933-s001.zip › Supplemental S2/PDBs_Match/EPHA2.png]

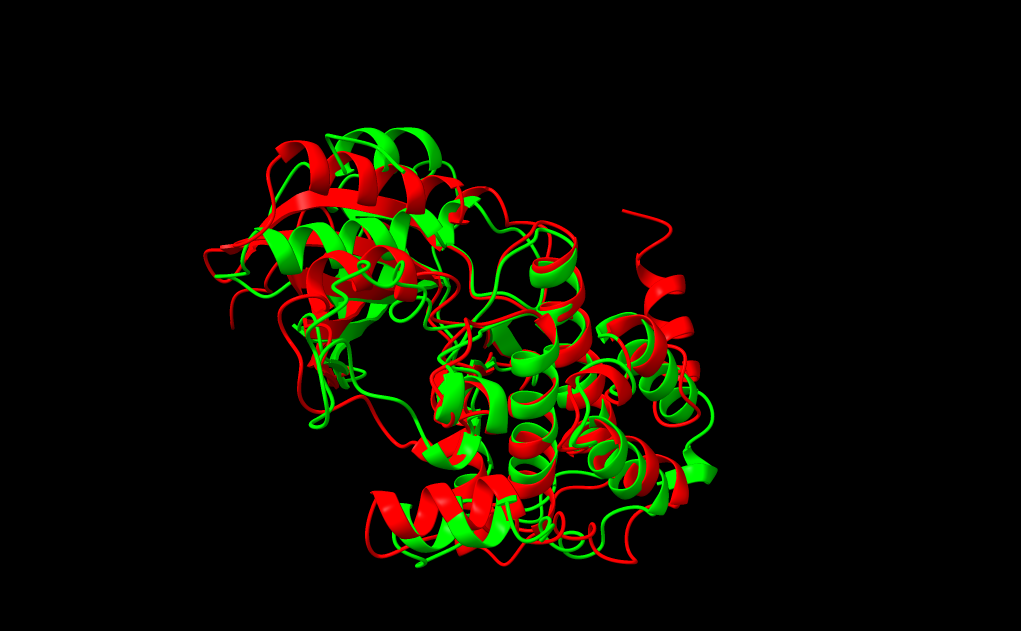

Supplement: Supplementary file 1 [file biomolecules-14-00933-s001.zip › Supplemental S2/PDBs_Match/EPHA4.png]

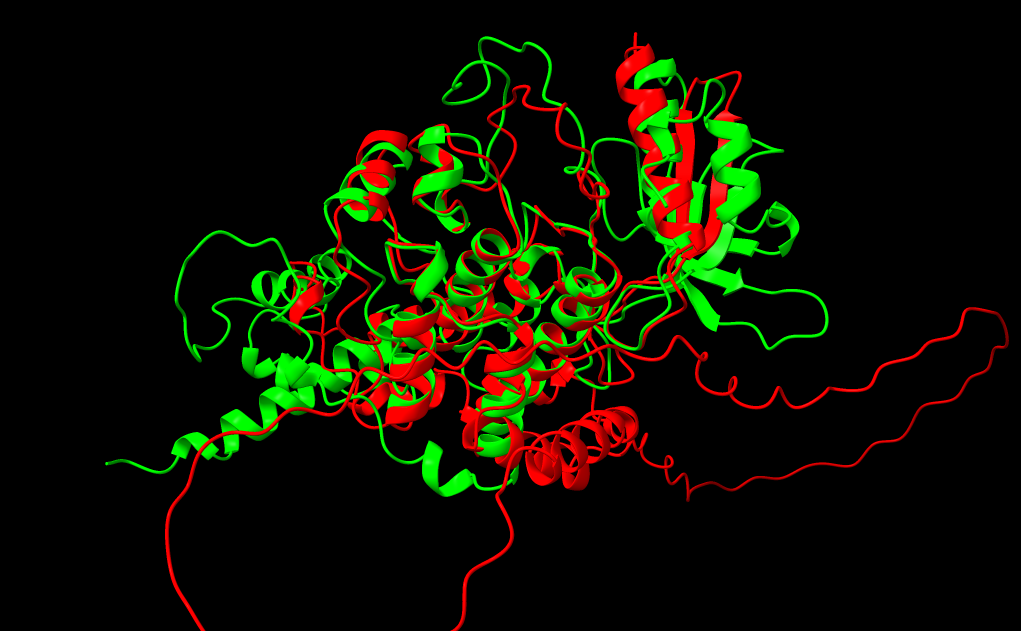

Supplement: Supplementary file 1 [file biomolecules-14-00933-s001.zip › Supplemental S2/PDBs_Match/EPHA5.png]

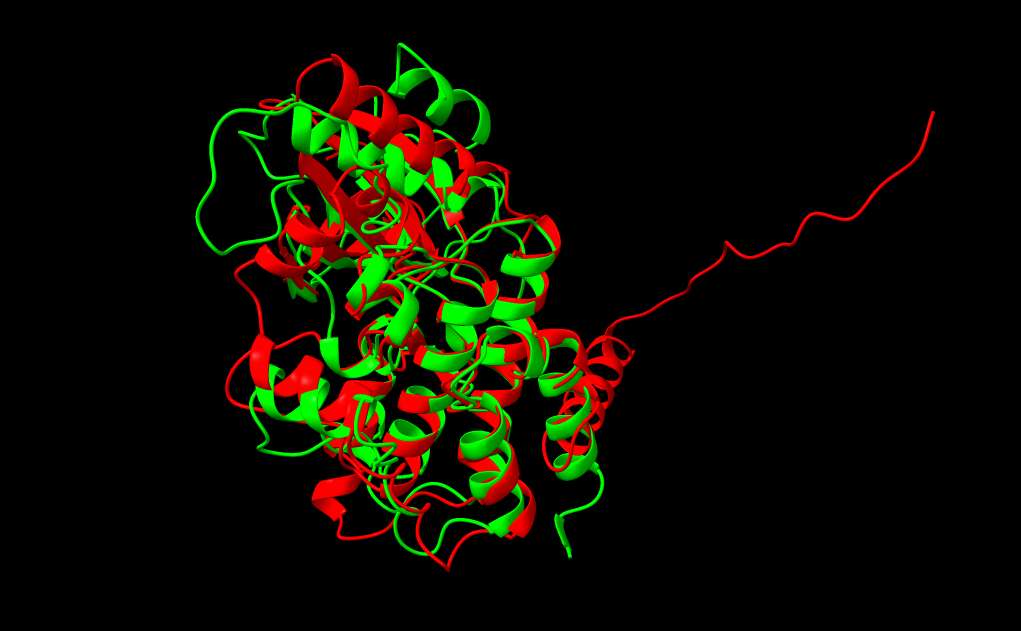

Supplement: Supplementary file 1 [file biomolecules-14-00933-s001.zip › Supplemental S2/PDBs_Match/EPHB2.png]

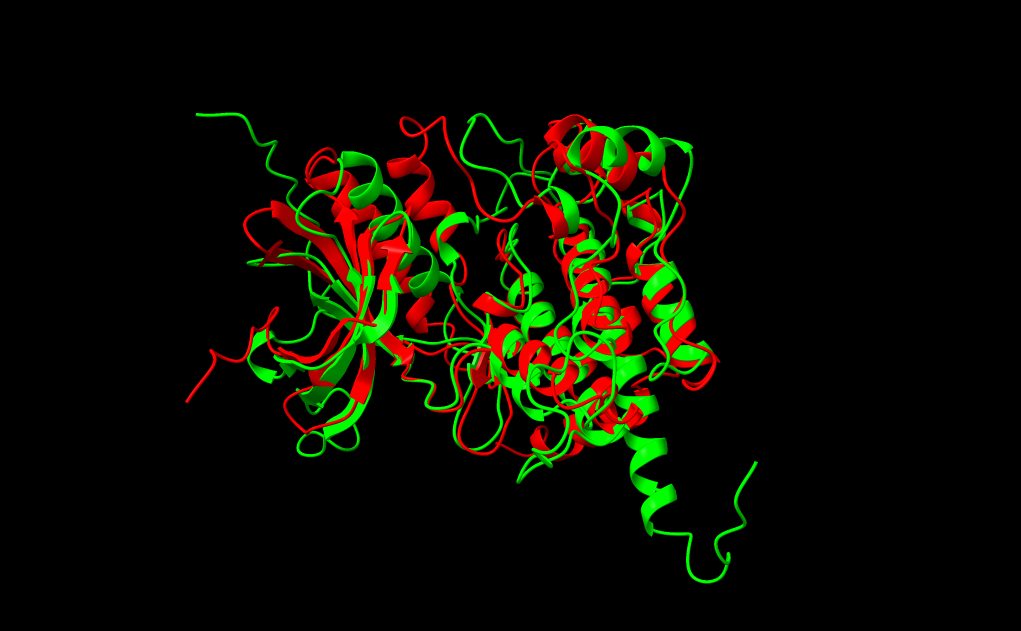

Supplement: Supplementary file 1 [file biomolecules-14-00933-s001.zip › Supplemental S2/PDBs_Match/FGFR2.png]

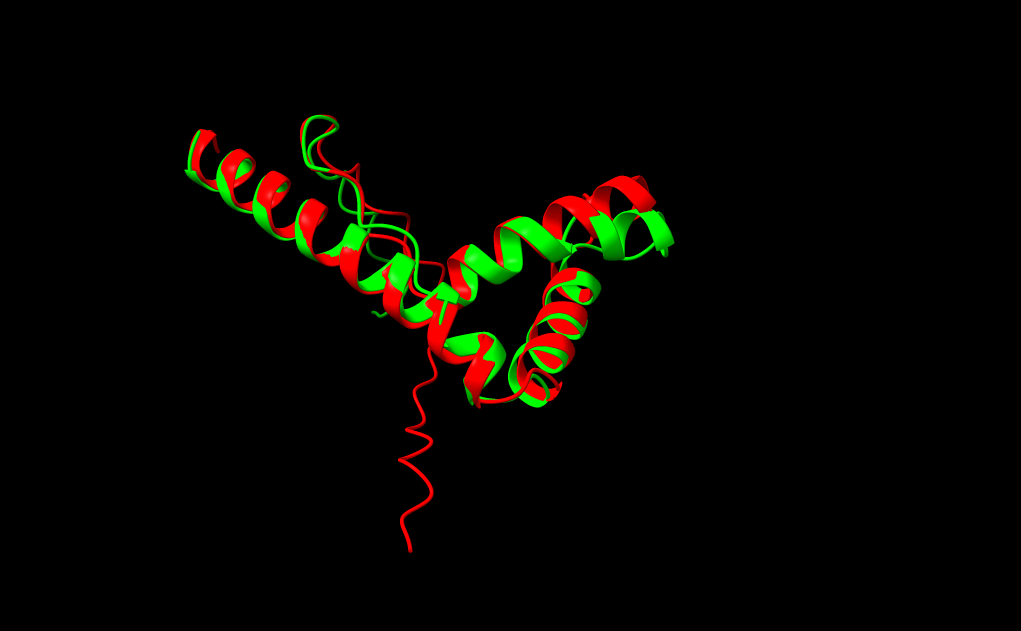

Supplement: Supplementary file 1 [file biomolecules-14-00933-s001.zip › Supplemental S2/PDBs_Match/HMGB1.png]

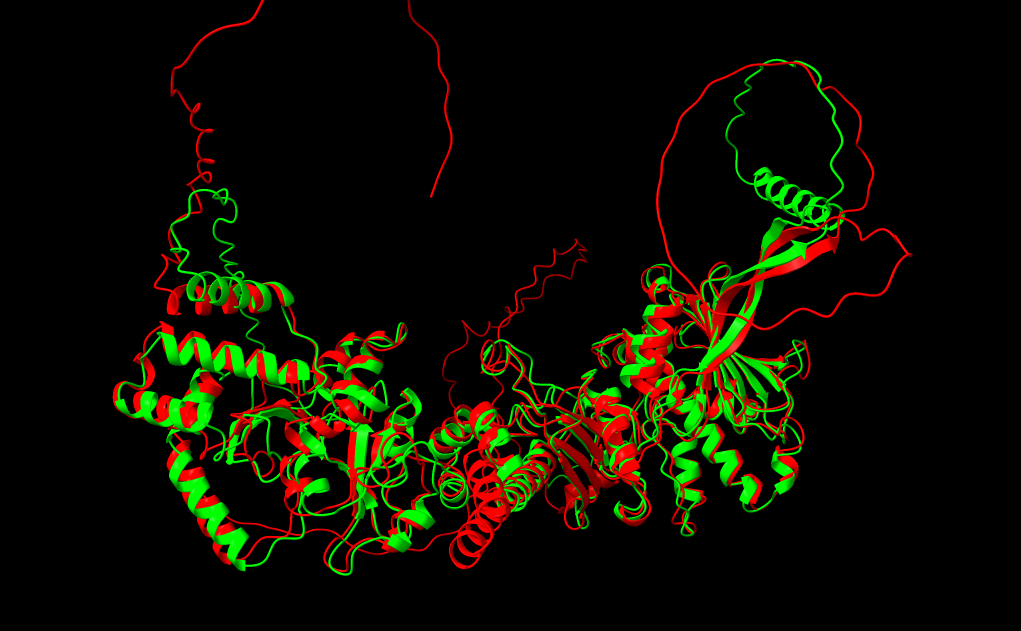

Supplement: Supplementary file 1 [file biomolecules-14-00933-s001.zip › Supplemental S2/PDBs_Match/HSP90AA1.png]

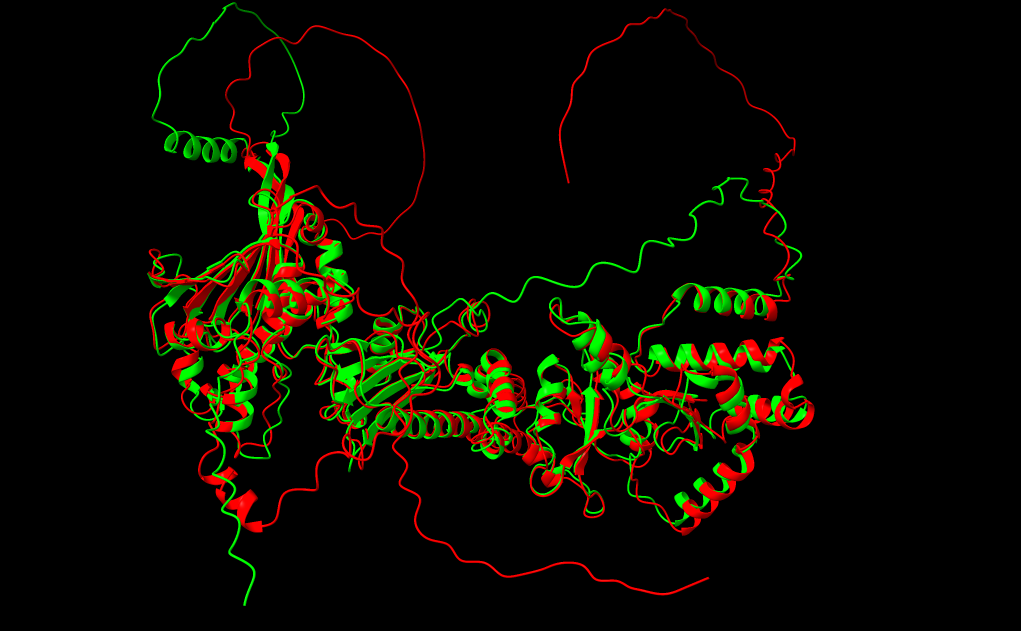

Supplement: Supplementary file 1 [file biomolecules-14-00933-s001.zip › Supplemental S2/PDBs_Match/HSP90AB1.png]

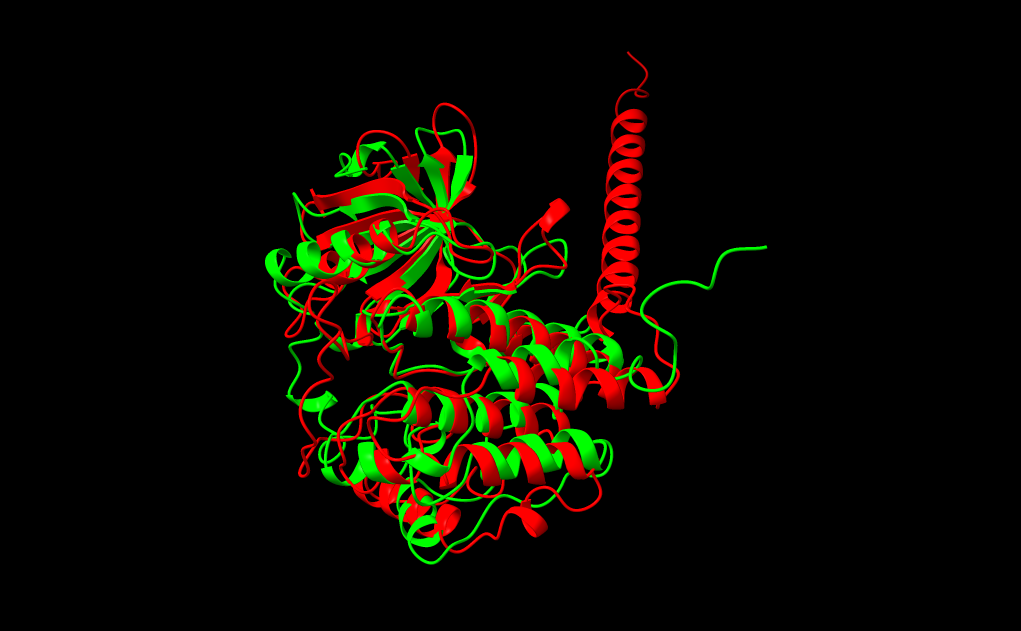

Supplement: Supplementary file 1 [file biomolecules-14-00933-s001.zip › Supplemental S2/PDBs_Match/LYN.png]

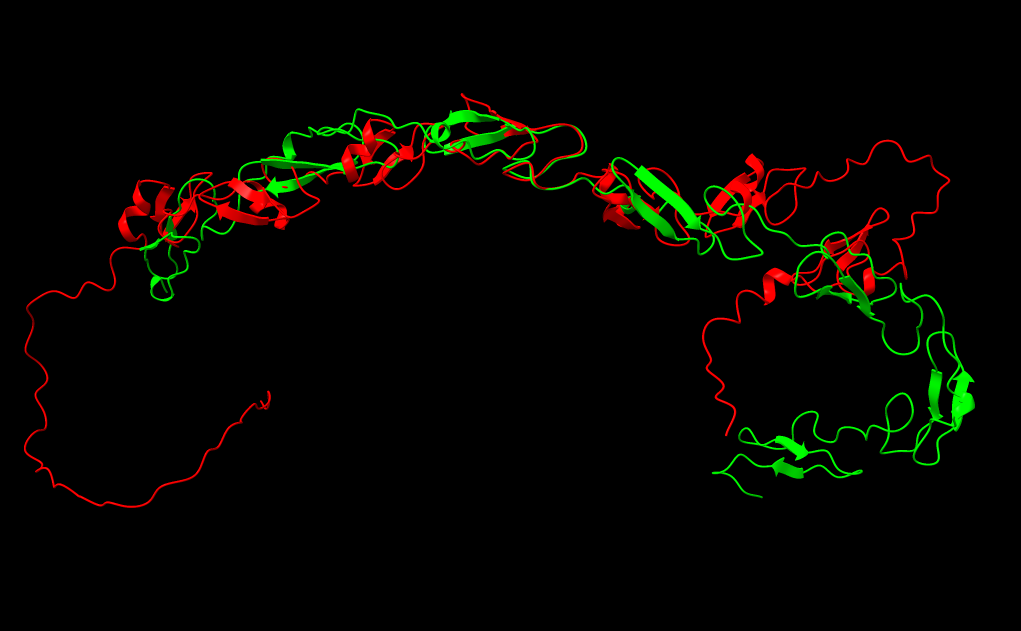

Supplement: Supplementary file 1 [file biomolecules-14-00933-s001.zip › Supplemental S2/PDBs_Match/NOTCH1.png]

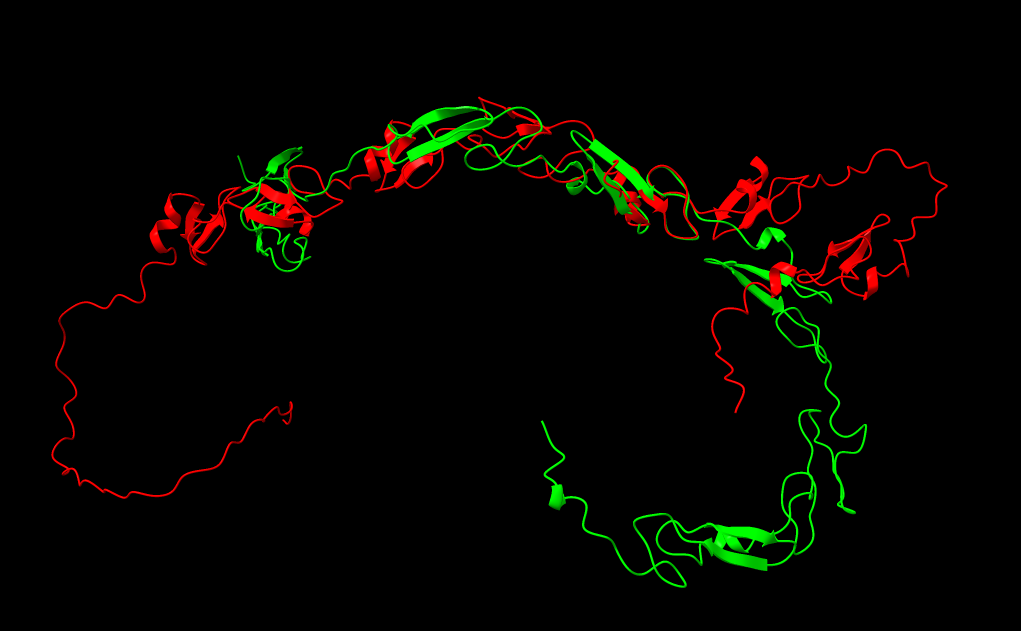

Supplement: Supplementary file 1 [file biomolecules-14-00933-s001.zip › Supplemental S2/PDBs_Match/NOTCH2.png]

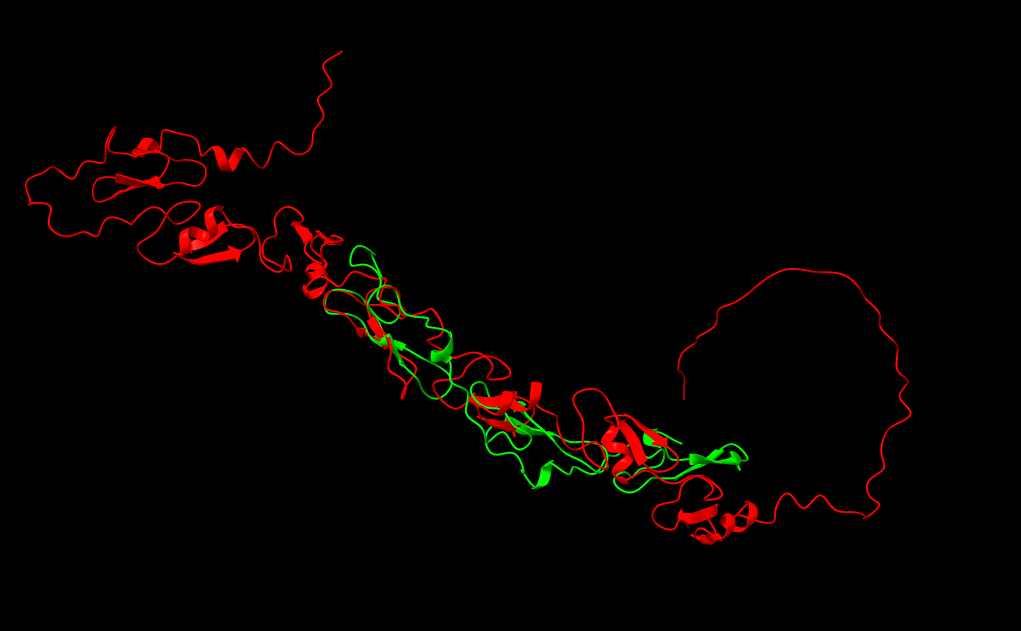

Supplement: Supplementary file 1 [file biomolecules-14-00933-s001.zip › Supplemental S2/PDBs_Match/NOTCH3.png]

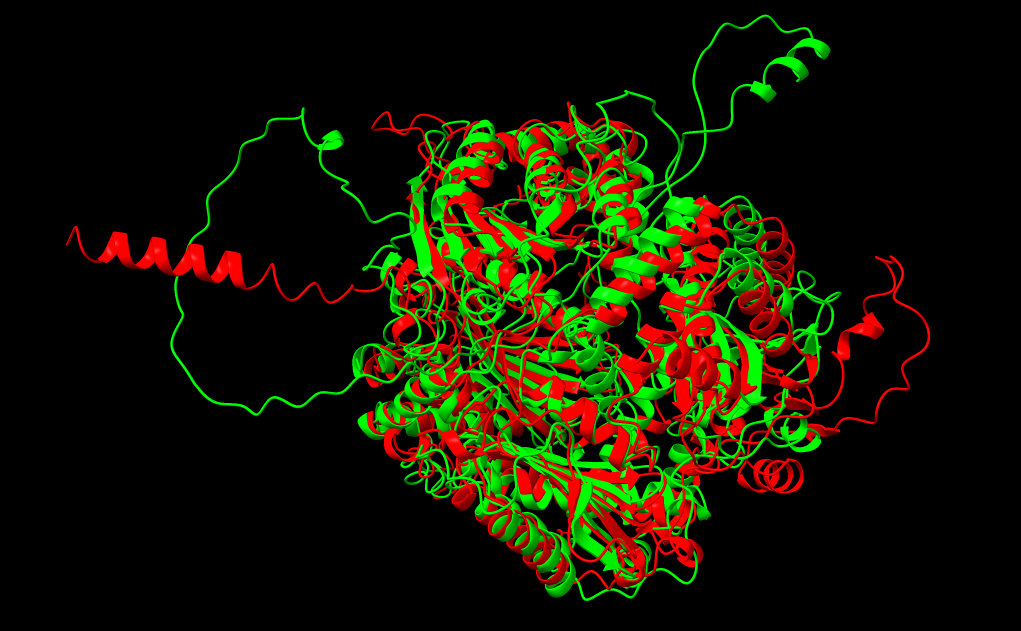

Supplement: Supplementary file 1 [file biomolecules-14-00933-s001.zip › Supplemental S2/PDBs_Match/NRDC.png]

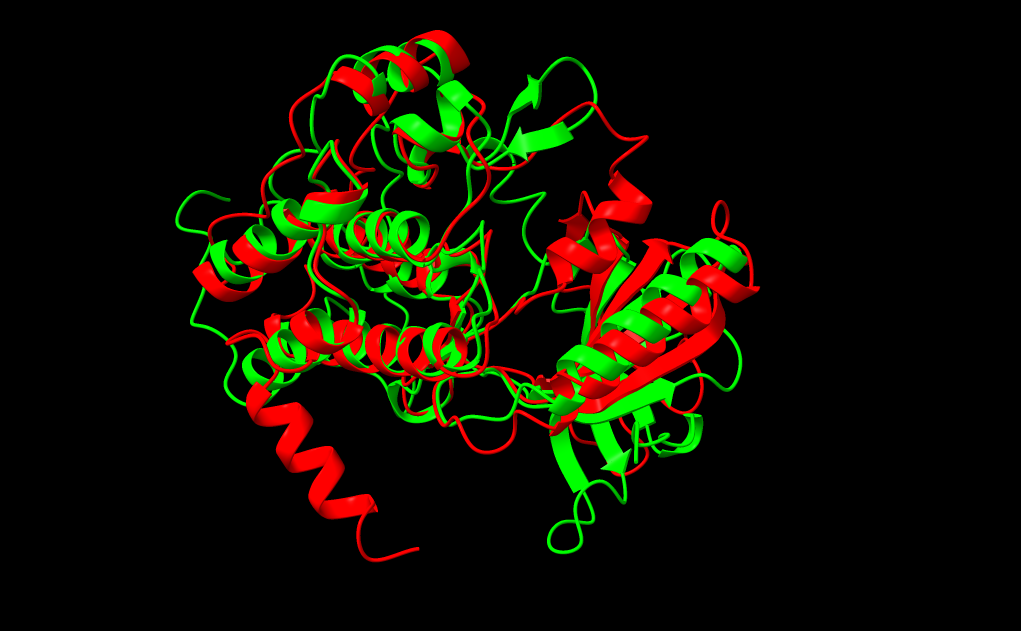

Supplement: Supplementary file 1 [file biomolecules-14-00933-s001.zip › Supplemental S2/PDBs_Match/NTRK1.png]

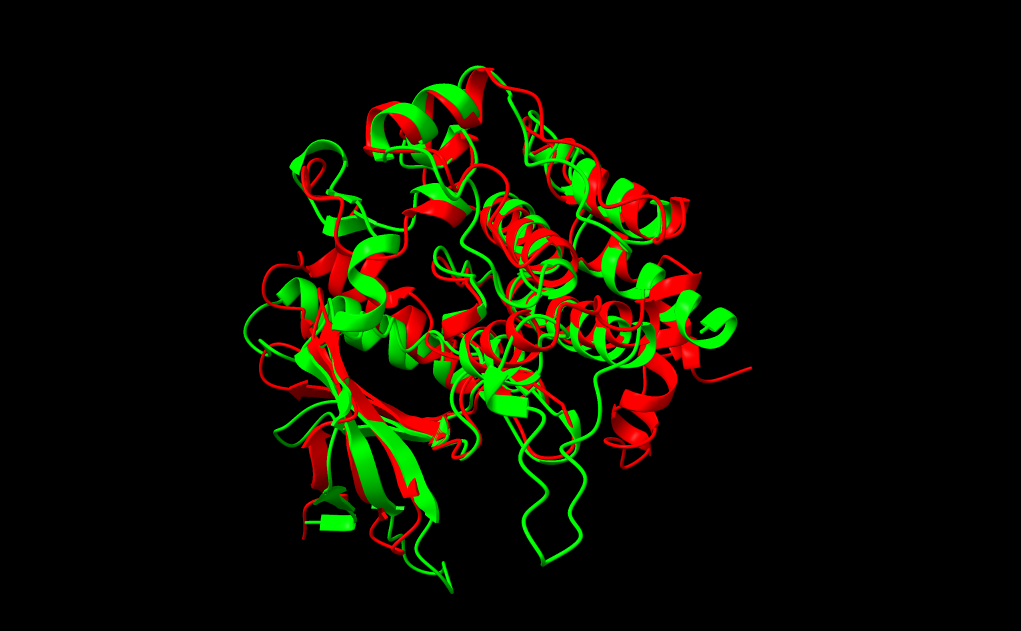

Supplement: Supplementary file 1 [file biomolecules-14-00933-s001.zip › Supplemental S2/PDBs_Match/ROR1.png]

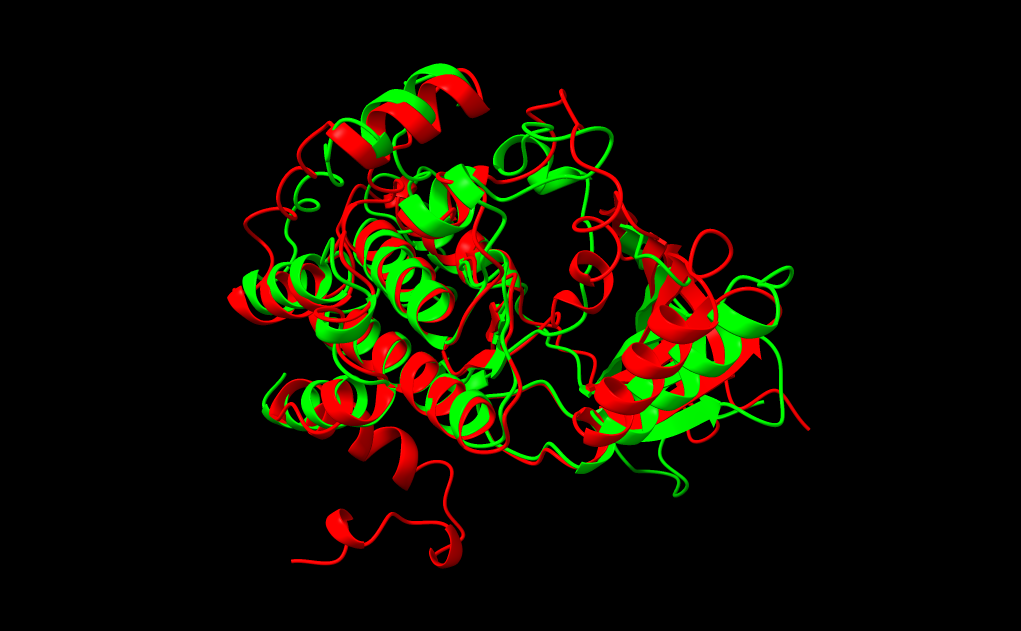

Supplement: Supplementary file 1 [file biomolecules-14-00933-s001.zip › Supplemental S2/PDBs_Match/ROR2.png]
